# Green Purchase Behaviour Gap: The Effect of Past Behaviour on Green Food Product Purchase Intentions among Individual Consumers

**DOI:** 10.3390/foods13010136

**Published:** 2023-12-30

**Authors:** Lucyna Witek, Wiesława Kuźniar

**Affiliations:** 1 Department of Marketing, The Faculty of Management, Rzeszow University of Technology, 35-959 Rzeszów, Poland; 2 Department of Marketing and Entrepreneurship, Institute of Economics and Finance, University of Rzeszow, 35-959 Rzeszów, Poland; wkuzniar@ur.edu.pl

**Keywords:** consumer, green purchase, green purchase behaviour gap, green food, TPB, past behaviour, green marketing, sustainable development, consumer’s attitude

## Abstract

The purpose of this study is to examine factors affecting green food product purchase intentions and, specifically, to specify the role of past behaviour in shaping purchase intentions for these products and in switching to environmentally friendly food purchases. As for the theoretical framework, the Theory of Planned Behaviour (TPB) was used, with certain modifications proposed; namely, additional constructs were included: past behaviour, knowledge, and trust in green food. Data were collected from 650 green product consumers in Poland. The online survey method was employed. The research revealed that past behaviour is a powerful indicator, which, to the greatest extent, explains purchase behaviours towards green food. This paper explores the fact that attitudes are also strongly linked to green food purchase intentions. Moreover, social norms, trust, and knowledge also have a positive effect on the intention to purchase green food products. Perceived behavioural control is relatively weak but statistically significant. The extended model explains 57% of the variance in green food purchase intentions. By incorporating past behaviour into the TPB, this study gives a new insight into understanding the inconsistency between positive attitudes towards green food and real purchase behaviours. The results of the study provide managers working in the food sector with relevant guidelines for the design of marketing strategies.

## 1. Introduction

In recent years, sustainable development has become an important topic, discussed globally by both practitioners and scientists. The awareness of processes taking place in the global economy changes market entities’ perception of production, consumption, products, and marketing. Consumers use the environment and are more and more concerned about its degradation. Consumers’ switching to green products may help minimise adverse environmental effects and benefit health. Consumers buy organic food because they consider it safer than conventional food [1]. This is due to the absence of toxins and harmful chemicals such as fertilisers or pesticides. Some consumers are convinced that green food is more nutritious than conventional food and see health benefits for themselves from consuming it [2].

The awareness of the negative consequences of the consumption and purchase of products for our planet does not translate into eco-friendly consumer purchase behaviours. There is an inconsistency between their positive attitudes towards green food products and their purchases [3,4].

Many studies [5,6,7] have confirmed positive attitudes towards the purchase of green products and their impact on the intention to consume or purchase green products; however, some researchers [8,9,10,11] point to green purchasing inconsistencies.

In order to better understand the gap between consumers’ attitudes and behaviours, also in the green food product market, the modification of the TPB was proposed. Previous research [12,13] identified weaknesses in the TPB and proposed modifications to the model. Incorporating additional variables into the TPB model enhanced its predictive power [14,15].

The present study proposes the extension of the TPB to include other variables, which are designed to improve its efficiency in predicting consumers’ behaviour based on intentions. Predicting their intentions to a better degree may ensure that past behaviour, knowledge, and trust are taken into consideration. Such an approach seems to be reasonable, as research by Yadav and Pathak [16] proved the effectiveness of the extended TPB as a research model in explaining consumers’ intentions regarding the purchase of green products. An analysis of 232 other studies was also performed by Sharma [17], in which past behaviour, knowledge, and trust were identified as a few of the key factors behind consumers’ green purchase behaviours. Haws et al. [18] observed that consumers’ future behaviour towards green products is predicted on the basis of past behaviour, Costa et.al. [19] relying on the Theory of Planned Behaviour, show that attitudes play a role as the antecedent of green product purchase intentions and that a variable does not mediate the effect of previous purchase experiences on purchase intentions.

This article aims to provide a deeper understanding of factors affecting green food purchase intentions and, specifically, to specify the role of past behaviour in shaping purchase intentions for these products and in shifting to eco-friendly purchases. The article also expands the knowledge for understanding how the green-attitude–behaviour gap occurs and is minimised.

This study provides a basis that decision-makers may rely on to find out how to transform consumers’ concerns over environmental pollution into real eco-friendly purchase behaviours. The study indicates—and this can be of relevance, in particular, to marketing managers—directions for the development of marketing strategies and how customers’ experiences can be created in green food marketing to trigger trial purchases, offer purchase experiences, and build up trust, which will translate into higher demand for green food and other eco-friendly products in the future. The conceptualisation of factors and development of a framework based on the theoretical context and empirical research explaining consumers’ purchase behaviours towards green food products can help stimulate consumers’ environmentally responsible purchasing processes in the future.

## 2. Theoretical Framework and Hypotheses

The Theory of Planned Behaviour (TBP), put forward by Ajzen, established a theoretical framework for predicting and understanding consumer behaviour [20]. The theory takes into account three factors (attitude, subjective norms, and perceived behavioural control) and integrates them into a conceptual model [21]. The theory implies that consumers’ behaviours are clear expressions of their intentions. Many studies have relied on the TPB to predict intentions for green product consumption and green purchases [22,23] , proving its usefulness and soundness. The usefulness of the Theory of Planned Behaviour on the food market was examined by research [24,25,26].

Behavioural intention is a basic variable in the TPB, being an important predictor of real behaviour [27]. Intention constitutes declared readiness to behave in a given manner, which is associated with the effort that consumers are willing to undertake with a view to making a purchase. Intention indicates to what extent consumers will strive to purchase green food products. Many studies have demonstrated that intention plays a pivotal role in mediating the transition between an attitude towards purchasing a green product and its purchase [12,14,28,29].

The intention to purchase a green product is linked to the attitude towards that purchase, which is rooted in a consumer’s conviction about the expected consequences of the purchase. An attitude towards green food results from a consumer’s behavioural convictions and an assessment of the purchase, leading to a conviction about the possible implications of such purchasing behaviour. An attitude towards green food product purchases stems from a consumer’s conviction about the benefits and consequences of the purchase and from the values attributed to them. A consumer will choose green food that has the greatest subjective usefulness. The higher the subjective value of the expected outcome of a purchase, the more positive the attitude towards that purchase and, consequently, the stronger the intention observed. Many studies [19,30,31] have confirmed the positive effect of attitudes on intentions to purchase a green product. This served as the basis for Hypothesis 1, which states that attitudes towards the purchase of green food products have a positive effect on purchase intentions.

**Hypothesis** **1** **(H1).**  *Attitudes towards purchasing green food products (AGFP) have a positive effect on the intentions to purchase green food products (IGFP).*

Social norms specify how other consumers, e.g., family members, friends, and workmates, influence a consumer’s behaviour. As far as the purchase of a green product is concerned, social norms are associated with a consumer’s conviction that people whom he or she considers important will accept his or her decision to purchase a green product. Some studies [14,31] provide evidence showing that social norms do not act as a direct antecedent of purchase intentions, whereas other research (e.g., [29,32] demonstrates that social norms have a profound effect on the intentions to purchase green products. Hence, this leads to the hypothesis that social norms have an effect on green food product purchase intentions.

**Hypothesis** **2** **(H2).**  *Social norms (SN) have a positive effect on the intentions to purchase green food products (IGFP).*

Perceived behavioural control reflects the individual’s perception of how easy or difficult it is to purchase a green product. It can be associated with situational factors such as economic costs, the availability of information about the product, the product availability, or the convenience and ease of use. It is very unlikely that consumers will engage in purchasing green food products if they believe that making the purchase is difficult or that the effort related to the purchase of a green food product does not bring any benefits to the purchaser or the environment. Many studies [12,33,34] have found that perceived behavioural control has an effect on the intention to purchase a green product. Onel [35] demonstrated that perceived behavioural control is of no relevance to the explanation of this behaviour. In light of these findings, it was hypothesised that perceived behavioural control is of no relevance to the explanation of green food product purchases. Hence, the following hypothesis was formulated, namely, that perceived behavioural control does not have any effect on green food product purchase intentions.

**Hypothesis** **3** **(H3).**  *Perceived behavioural control (PBC) has no effect on the intentions to purchase green food products (IGFP).*

Trust is a relevant factor behind consumers’ purchase decisions. It is related to convictions and expectations about the eco-friendly properties of products, their accurate marking, their reliable warranty, and the manufacturer’s green declarations. Trust serves as a measure of customers’ convictions about the environmental and health effects of green food purchases. A purchaser is not able to predict or assess all of the consequences arising from a purchase. Many studies, e.g., Wang et al. [36] have shown that trust has an effect on the intentions to purchase green products. Nuttavuthisit and Thøgersen [37] referred to a lack of trust, which decreases the consumer’s perception of the benefits of purchasing green food and makes them less willing to pay more and buy green food. A factor that diminishes trust in green products is greenwashing [38,39]. Thus, the following hypothesis is proposed: trust in green food products (TGFP) has a positive effect on the intentions to purchase green food products (IGFP).

**Hypothesis** **4** **(H4).**  *Trust in green food products (TGFP) has a positive effect on the intentions to purchase green food products (IGFP).*

Trust in green products may not, however, be perceived as the only factor, as it is combined with other concepts, including knowledge and past behaviour. Knowledge is an important factor that drives the decision to purchase a green product [40]. Most consumers have some knowledge of green food products, yet they do not necessarily rely on it when making a purchase [36]. Mohd Suki [41] arrived at the conclusion that the knowledge of green brands is the most significant determinant of green product purchase intentions. Therefore, the following hypothesis was put forward, namely, that the knowledge of green food products has a positive effect on the intentions to purchase green food products.

**Hypothesis** **5** **(H5).**  *The knowledge of green food products (KGF) has a positive effect on the intentions to purchase green food products (IGFP).*

Past behaviour constitutes a significant predictor of future behaviour. Past behaviour is linked to consumers’ direct experiences regarding products purchased in the past and has an effect on consumer awareness [42]. Empirical research [43] verified that, in addition to attitudes, social norms, and perceived behavioural control, past experiences also significantly predict intentions to purchase natural products. Also, the findings revealed by Pakpour et al. [44] demonstrated the direct effects of not only perceived behavioural control, subjective norms, and attitudes but also past behaviour on green product purchase intentions. Past behaviour towards the purchase of green apparel had an effect on the perception of the benefits of green apparel and, consequently, on the purchasing of apparel [45]. Hence, the following hypotheses were formulated:

**Hypothesis** **6** **(H6).**  *The greater the past behaviour towards purchasing green food products (PB) is, the stronger the intentions to purchase green food products (IGFP) are.*

**Hypothesis** **7** **(H7).**  *The intention to purchase a green food product is a function of the attitude towards the purchase of green food products, social norms, past behaviour, trust in green food products, and knowledge of them.*

**Hypothesis** **8** **(H8).**  *Past behaviour (PB) explains, to the greatest extent, the intentions to purchase green food products (IGFP).*

To verify the hypotheses, a series of variables needed to be measured. The process of identifying the factors explaining purchase behaviours towards green food products used the TPB, which determined the selection of variables and their measurement. Items were constructed based on literature sources (Table 1).

**Figure 1:**
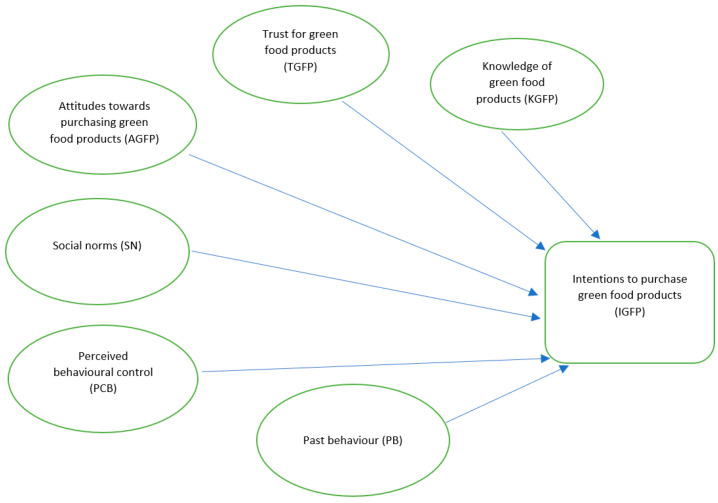
Proposed theoretical framework.

To define the model, the following measures were developed: attitudes towards purchasing green food products (AGFP), social norms (SN), perceived behavioural control (PCB), trust in green food products (TGFP), knowledge of green food products (KGFP), and past behaviour (PB). They were used as independent variables, whereas it was assumed that the intentions to purchase green food products (IGFP) would serve as a dependent variable (Figure 1).

To deploy the model, the TPB assumptions had to be considered. For the assessment of the AGFP variable, attitudes towards the purchase of a green product and its benefits relating to environmental protection, health, safety, and high quality were taken into account. As regards the SN variable, the influence of family and friends was deemed the most important referent, whereas the PCB construct was evaluated only on the basis of the following factors: income, time, price, habits, and the ease of being found. The TGFP variable included consumers’ trust in green food manufacturers and the high quality of their products, as well as consumers’ trust in eco-friendly manufacturing methods. The assessment of the KGFP variable relied on the respondents’ subjective evaluation concerning their knowledge of green food, manufacturing methods, marking, and availability. PB was measured by the frequency of purchasing green food in the past and the degree of satisfaction with green food purchased in the past. The period of planned behaviour was set to cover three months, starting at the completion of the study.

The reliability of the measurement scales used was tested with Cronbach’s alpha. The scale used for attitudes towards the purchase of a green product demonstrated the highest reliability (Cronbach’s alpha equal to 0.9). The other components had equally satisfactory values for the said coefficient, namely, 0.7–0.81. The acceptable values of alpha range from 0.70 to 0.95 [56].

## 3. Research Methodology

The survey questionnaire was prepared according to ethical standards. Potential human participants in this online survey study could decide whether to take part in the study or not. The survey study covered the people who filled in the questionnaire. Before candidates completed the survey questionnaire, they had read details about the survey study. The survey was conducted anonymously and on a voluntary basis. Data were only obtained to assess the characteristics of the population at a given time. The questionnaire was prepared based on Regulation (EU) 2016/679 and the Polish Legislation—The Personal Data Protection Act of 10 May 2018. Anonymity was guaranteed for all participants in the survey research.

Before the study participants completed the questionnaire, the candidate study participants were asked a recruiting question: “Have you bought green food in the last 3 months?”. Then, they moved on to the main questionnaire.

The questionnaire consisted of three parts. The first part of the questionnaire included information about the survey, its author, and affiliation. In addition, it included information about the anonymity of the study and the estimated time of the study. It was estimated that the time needed for a consumer to complete the survey was ten minutes. Participants were informed about the purpose of the survey. The aim of the survey was to examine the attitudes and behaviours of purchasers in the green product market. Additionally, respondents were instructed to answer the questionnaire questions based on their own viewpoints or personal behaviour in their respective areas. A brief introduction explained the length of the questionnaire. The second part of the questionnaire consisted of two sections. The first section collected data on consumption and purchasing habits in the green product market. The second section contained 32 statements. Participants are asked whether they disagree or agree with a particular statement. These items were evaluated by the respondents on a seven-point Likert scale, where 1 = strongly disagree and 7 = strongly agree. The Likert scale has been used effectively in previous studies [57,58].

The third part of the questionnaire contained information about the socio-demographic characteristics of the respondents, including age, gender, place of living, and number of children. The survey questionnaire was prepared in Polish. The reliability of the questionnaire was guaranteed through the administration of a pilot study conducted among 30 consumers to eliminate errors and inaccuracies.

Data were collected online from 826 surveys, but some questionnaires were eliminated due to missing responses or sample characteristics. The analysis covered 650 Polish consumers who declared that they had purchased green food products over the past three months. Candidates who met the criteria for participating in the study received a link to the survey. The link was sent in an invitation using social media and an email with the invitation by trained volunteers living in various parts of Poland.

The respondents’ age, place of residence, and gender were used as controlled variables. As for age, the sample structure corresponded to the structure of the Polish population who have access to the Internet. The distribution of the respondents was consistent with the proportions in the structure of the Polish population by place of residence. The gender structure was as follows: 70% women and 30% men. Women are more frequently responsible for purchasing foodstuffs for their households than men. Moreover, previous research has shown that they purchased green food more often than men [59,60]. As regards the educational backgrounds of the respondents, they answered that they completed higher education (61%), secondary education (31%), or vocational and lower education (8%). The following social and demographic characteristics were exhibited by the research sample: gender, age, place of residence, and number of children (Table 2).

The study was carried out for one month and was completed in January 2019. Due to its low cost and effectiveness of data collection, the online survey method was employed. A lot of research dealing with the examination of consumer behaviour employs a survey as a method, ensuring the possibility of reaching purchasers and enabling the research to be carried out in a relatively short time [61,62]. Furthermore, an online survey leads to the low cost of research work and is effective in studying intentions in the green food market [63,64].

The empirical material of the results was analysed using the IBM SPSS Statistics 27.0 statistical package. 

## 4. Results

This study incorporates attitudes (Table 3), social norms (Table 4), and perceived control of behaviour (Table 5) as constructs and trust (Table 6), knowledge (Table 7), and past behaviour (Table 8) as additional constructs, which can help explain green food product purchase intentions (Table 9), consequently giving a better view of how the green purchasing gap is understood and created. The model included attitudes, social norms, perceived control of behaviour, past behaviour, trust, and knowledge as independent variables, as well as a dependent variable—intentions.

The variables selected for the model are linearly correlated with each other, which is reflected by correlation coefficients. The correlations between the variables are presented in Table 10. By analysing Pearson’s correlation coefficients, which show the relationships between the features, one may claim that attitudes towards purchasing green food products (AGFP) have a positive effect on the intentions to purchase green food products (IGFP). The dependent variable—purchase intentions—is strongly linked to the variable concerning attitudes (0.60). The more positive the attitude towards green products, the greater the willingness to purchase them. Hence, Hypothesis 1 is supported.

Social norms (SN) also have a positive effect on the intentions to purchase green food products (IGFP) (0.5). The study suggests the importance of not only positive attitudes towards green food but also the impact of the social environment, such as family, peers, friends, or mates. Hence, Hypothesis 2 is supported. The opinions of people around a person were significant for his or her purchases, although to a lesser degree than attitudes.

The relationship with perceived behavioural control (PCB) is relatively weak but statistically significant (0.21). The greater the consumer’s control over a purchase, that is to say, the more smoothly such purchasing proceeds and the more feasible it is, the greater the willingness displayed by the consumer. The foregoing gives grounds for rejecting Hypothesis 3, which suggests that perceived behavioural control (PBC) has no effect on the intentions to purchase green food products (IGFP).

This study demonstrates that trust in green food products (TGFP) has a positive effect on the intentions to purchase green food products (IGFP). Hence, Hypothesis 4 is supported. By the same token, the knowledge of green food products (KGFP) has a positive effect on the intentions to purchase green food products (IGFP). Hence, Hypothesis 5 is supported.

The dependent variable, IGFP, is related to the greatest extent to the variable PB (0.61). The greater the green product or food purchase experience one has, the stronger the intention to purchase such a product; therefore, Hypothesis 6 is supported.

The measures to be included in the model (as independent variables) were checked to verify that the interconnection between them was not too strong. As there were no very significant relationships between the independent variables, all of them could be incorporated into the model.

The forward stepwise regression method was employed to determine which variables, and in which order, entered the model and described the variable IGFP to the biggest extent. The first variable to enter was PB (R2 = 0.37). Another step enabled the next variable to be added to the model, namely, attitudes towards purchasing green food products (AGFP) (R2 = 0.52), followed by SN (R2 = 0.54) and KGFP (R2 = 0.56). The feature corresponding to trust was the last significant variable that entered the model (R2 = 0.57). The other features were not statistically significant. Perceived behavioural control did not enter the model. The results of the estimation of model parameters are shown in Table 11.

The model fits the empirical data and explains 57% of the variance. It is characterised by a normal distribution of residuals, and hence, it has been constructed correctly (*p* = 0.62506).

The intention to purchase a green food product is a function of the attitude towards the purchase of green food products, social norms, past behaviour relating to purchases, trust in green food products, and knowledge of them. Hence, Hypothesis 7 is not supported.

The results of the study revealed that the most significant factor that explains the purchaser’s behaviour towards green food products is past behaviour related to experiences regarding the consumption and purchase of green food products. Therefore, past behaviour (PB) explains, to the biggest degree, the intentions to purchase green food products (IGFP). Hence, Hypothesis 8 is supported.

## 5. Discussion

Incorporating additional variables, such as past behaviour (PB), knowledge (KGFP), and trust (TGFP), into the proposed model demonstrated that the previous behaviour that a consumer has already had with the purchasing of green food products and positive attitudes play the most prominent roles. The richer the experiences with green food product purchases that one has, the more favourable the attitude that he or she displays, the greater the significance gained by social norms, knowledge, and trust, and the higher the likelihood that a purchase will be made in the future. An increase in experience leads to a growth in knowledge and results in the risk of the purchase being perceived as lower, which, consequently, translates into the intention to repeat the behaviour in the future. The modified model explained 57% of the variance in green product purchase intentions. The model incorporating only the variables covered by the TPB explained 45% of the variance in green food product purchase intentions [65]. The addition of past behaviour relating to product purchases to the model improved the degree to which the model fit the empirical data and enhanced the effectiveness of the research instrument being developed. The determination index stood at 0.37, meaning that 37% of the variance in purchase intentions explained past behaviour towards the purchase of a green product. The significance of past behaviour was confirmed by St Quinton [66], who saw a considerable variance increase after incorporating experience into the TPB. Past behaviour has a positive effect on the green intentions of consumers and constitutes an important predictor of future behaviours towards green purchases, which is in line with the findings of [54,67,68].

This research showed that attitudes affect the intentions to purchase green food products. An attitude towards a purchase stems from a consumer’s convictions about the benefits and features of green food products and the values attributed to them. Consumers must appreciate environmental protection before they intend to purchase environment-friendly food products. The consumer will choose a product that has the greatest level of subjective usefulness. The greater the subjective value of the expected outcome of the green food product purchase, the more positive the attitude towards such behaviour. This is consistent with research carried out by [69,70,71].

The social sphere may affect consumer behaviour towards green food. These findings are empirically exemplified by the research performed by [72,73]. Consumers who perceive green products in a positive way have an impact on the shaping of other consumers’ attitudes. Attitudes towards green food purchases, whether positive or negative, spread among people [74] Research of [75] pointed to the fact that influencers play a prominent role in the shaping of the eco-friendly behaviours and attitudes of Generation Z.

Consumer knowledge of the rules for the environment-friendly conduct, features, and eco-labelling of food, the awareness of benefits of the consumption and purchasing of green food products, and the skills required to find them at the point of sale were significant predictors of purchase behaviours towards green food [76]. Knowledge is also the factor that reinforces a consumer’s personal conviction about having greater control over green product purchases [77,78]. People who have knowledge of such matters as features, benefits, and marking were more willing to pay a higher price for green food than those lacking that knowledge [79].

A purchaser may take a positive attitude towards purchasing an environment-friendly product; however, he or she may have no intention to behave in a specific manner, especially when difficulties are seen by him or her. Hence, the conviction about control is connected with the consumer’s conviction about factors that can either facilitate or hinder the purchase of green food products (e.g., time, money, availability).

## 6. Conclusions

The food industry has a powerful effect on the natural environment. In recent years, companies have been mounting a continuing effort to mitigate adverse impacts on the environment. A re-examination of these issues is necessary to determine the factors explaining consumers’ purchase intentions in the context of narrowing the gap between positive attitudes towards green food and its purchases. The enormous potential that the green food market has in Central and Eastern Europe gives reasonable grounds for researching these issues.

The study conducted provides theoretical and managerial contributions. It verifies the possibility of using additional constructs other than the TPB, such as past behaviour, knowledge, and trust in developing countries. As demonstrated by this study, incorporating additional variables into the model provides a sounder basis for the explanation of green food purchase intentions in the context of Central and Eastern Europe’s developing market. The proposed model is a useful instrument to examine the effect of switching to green purchases not only in Poland but also in other countries. It can be utilised to explain purchase intentions relating not only to green food but also to other green products. The findings on past behaviour that are presented in this study contribute to a better understanding of the attitude–behaviour gap.

The present study carries implications for management and the creation of environmentally responsible marketing strategies. As environmental pressure and awareness increase, many companies are implementing strategies and innovations to become more responsible by developing products that meet the requirements of environmentally aware consumers. Such companies are interested in finding determinants for green purchase behaviours with a view to adopting their green marketing strategies. Marketing strategies, specifically promotional campaigns, should strive not only to inform consumers or raise their awareness but also, first and foremost, to broaden purchase experiences and promote favourable attitudes among consumers towards the purchase and consumption of green food. Marketing activities focused on broadening purchasers’ experiences, which are carried out, specifically, at a local level, would be of benefit to eco-friendly product purchases, even if they were more expensive. Experiences with green food products not only make consumers develop ecological awareness but also facilitate their recognition of products and make their commitment to green purchases deeper, which has a positive impact on minimising environmental degradation.

The contribution provides a basis for solutions in companies that encourage consumers to actually purchase green food products. The general comprehension of the issues pertaining to green food, a poor understanding of the unique and complex nature of green food, and an insufficient ability to grasp the precise meaning of benefits accruing from green food product purchases and the manufacturing methods, certification, and eco-labelling of such products may result in a green purchase being postponed for some time or even not being made at all in favour of non-green purchases. In such a situation, price reductions, sales promotions, tastings, and experiential marketing are instruments that deserve attention in order to enhance the direct experience with a purchase. These instruments seem to be very efficient tools for encouraging consumers to taste and finally purchase green food, as well as repeating that purchase in the future. It is also worthwhile to link the creation of green food experiences to increasing the availability of green food and its visibility on shelves, which suggests the need to create visual experiences at points of sale. Offering experiences in a shopping environment and using sensory marketing may help stimulate green product purchases. As for young people, the convergence of physical and virtual experiences might prove to be indispensable.

Broadening the consumer green food purchase experience and promoting favourable attitudes among consumers towards the purchase and consumption of green food may considerably contribute to narrowing the gap between positive attitudes towards green food and purchase behaviours and may be highly conducive to enhancing the effect of switching to green purchases.

## 7. Limitations and Past Research

The extension of the findings and conclusions to the entire population is also limited by the method and sample used for the study. The study deepens the understanding of the gap between favourable attitudes towards green food and purchase behaviours, pointing to the key role of past behaviour. The attempt to explain the gap between the attitudes and behaviours of consumers towards green food products causes many difficulties, as the decision-making process is affected by a variety of individual, social, and situational factors. Furthermore, the present study incorporated respondents’ subjective assessments of their knowledge of green food.

By looking at consumer behaviours, one may discern signs of ambivalent attitudes, being a response to both positive and negative product-related aspects and leading to approach-avoidance conflicts. For green food, it can be a conflict between taste and environmental protection. However, research by [39] demonstrated that highly ambivalent attitudes towards green products decrease green purchase intentions. Future research must examine that aspect in relation to the shaping of green food purchase intentions. Attitudes are not the only precondition for purchase behaviours. Importance must be attached to competences and opportunities. If attitudes are presented as relatively constant judgement processes, then it appears more reasonable to use the term “competences”, which would ensure greater transparency. Such an approach can be adopted for future research. The present studies are based on consumers’ declarations, which may distort the actual state of affairs. Nevertheless, future research can take into consideration other relevant variables that were not the focus of attention in this study, for instance, the perceived value of green food, perceived risk, and willingness to pay higher prices for green food. Many consumers may have negative experiences with green food, for instance, worse taste, less attractive external appearance, or a short shelf life. Incorporating negative experiences with purchases and consumption into the study of green food purchase intentions could deepen an understanding of past behaviour.

## Figures and Tables

**Table 1:** Variables in the extended TPB model.

Constructs	Items	Cronbach’s Alpha	Adapted From
Attitudes towards purchasing green food products (AGFP)	AGFP 1. Purchasing green food products protects the natural environment. AGFP 2. When I buy green food products I am sure that I help protect my health. AGFP 3. When I buy green food products I am sure that I help protect my security. AGFP 4. I am sure that when I buy green food products, I buy products of higher quality.	0.90	[16,28,31]
Social norms (SN)	SN1. My family members buy green food products. SN2. My friends think that, I should choose green food products.	0.77	[24,35,46]
Perceived behavioural control (PCB)	PCB1. I can buy green food products even if they have a higher price. PCB2. I have the competence to search for green food products among others available in the store. PCB3. I have time to purchasing of green food products. PCB4. My current habits do not prevent me from purchasing green food products. PCB5. I have the income to buy green food products.	0.73	[29,47,48]
Trust for green food products (TGFP)	TGFP1. I trust producers to ensure high quality. TGFP2. I trust manufacturers for selling products that protect health and the environment. TGFP2. I trust to friendly production methods. TGFP3. I trust environmentally friendly methods in production.	0.92	[37]
Knowledge of green food products (KGFP)	KGFP 1. I have knowledge about green food. KGFP 2. I have knowledge of environmentally friendly production methods. KGFP 3. I have knowledge about certification and labeling of green food. KGFP 4. I have knowledge about the availability of green food.	0.81	[28,49,50,51]
Past behaviour (PB)	PB1. I have bought green food at least once a week in the last 3 months. PB2. I have extensive experience with purchasing for green food. PB3. I am satisfied with my past purchases of green food.	0.70	[52,53,54,55]
Intentions to purchase green food products (IGFP)	IGFP 1. I plan to buy green food products in the next 3 months. IGFP 2. I am sure that during my next purchasing I will buy an green food product, even if it has a higher price. IGFP 3. I am willing to pay a higher price for an green product. IGFP 4. I am willing to switch to the ecological version of the product, but if the price and quality are similar.	0.73	[22,28,29]

**Table 2:** Characteristics of the participants.

Metrics	Items	Percentage
Age	18–24	13%
	25–35	30%
	36–45	28%
	46–55	17%
	55 and more	12%
Gender	Male	30%
	Female	70%
Place of residence	Village	31%
	Town, up to 40 thousand	19%
	Town, from 40 thousand to 100 thousand	13%
	City, from 100 thousand to 500 thousand	12%
	City, above 500 thousand inhabitants	25%
Number of children	0	23%
	1	1%
	2	10%
	3–4	47%
	5 and more	19%

**Table 3:** Attitudes towards purchasing green food products—response rates (N = 650).

Statements/Scale *	1	2	3	4	5	6	7
Purchasing green food products protects the natural environment.	1.38%	3.23%	7.85%	20.77%	23.38%	20.77%	22.62%
When I buy green food products I am sure that I help protect my health.	2.62%	3.54%	10.62%	24.00%	27.69%	30.15%	2.62%
When I buy green food products I am sure that I help protect my security.	4.31%	18.00%	22.00%	26.00%	25.08%	4.31%	18.00%
I am sure that when I buy green food products. I buy products of higher quality.	2.00%	2.92%	6.77%	18.77%	27.23%	21.54%	20.77%

* Likert scale: (1) Strongly Disagree; (2) Disagree; (3) Somewhat Disagree; (4) Neither Agree Nor Disagree; (5) Somewhat Agree; (6) Agree; (7) Strongly Agree.

**Table 4:** Social norms—response rates (N = 650).

Statements/Scale *	1	2	3	4	5	6	7
My family members buy green food products.	13.85%	15.38%	19.38%	20.46%	15.69%	8.62%	6.62%
My friends think that. I should choose green food products.	15.38%	20.00%	24.92%	16.62%	9.85%	4.15%	15.38%

* Likert scale: (1) Strongly Disagree; (2) Disagree; (3) Somewhat Disagree; (4) Neither Agree Nor Disagree; (5) Somewhat Agree; (6) Agree; (7) Strongly Agree.

**Table 5:** Perceived behavioural control—response rates (N = 650).

Statements/Scale *	1	2	3	4	5	6	7
I can buy green food products even if they have a higher price.	22.31%	22.00%	20.92%	12.62%	8.62%	5.08%	8.46%
I have the competence to search for green food products among others available in the store.	12.15%	7.54%	13.38%	20.15%	18.92%	14.46%	12.15%
I have time to purchasing of green food products.	17.23%	3.54%	5.69%	9.54%	18.00%	12.46%	17.23%
My current habits do not prevent me from purchasing green food products.	3.69%	6.62%	15.08%	22.15%	16.15%	17.08%	19.23%
I have the income to buy green food products.	4.92%	7.23%	17.23%	18.77%	25.08%	21.85%	4.92%

* Likert scale: (1) Strongly Disagree; (2) Disagree; (3) Somewhat Disagree; (4) Neither Agree Nor Disagree; (5) Somewhat Agree; (6) Agree; (7) Strongly Agree.

**Table 6:** Trust in green food products—response rates (N = 650).

Statements/Scale *	1	2	3	4	5	6	7
I trust producers to ensure high quality.	6.15%	13.54%	18.46%	25.08%	19.85%	12.46%	6.15%
I trust manufacturers for selling products that protect health and the environment.	6.15%	11.85%	18.77%	22.00%	22.77%	13.54%	6.15%
I trust to friendly production methods.	6.77%	12.62%	16.77%	19.69%	20.62%	14.46%	9.08%
I trust environmentally friendly methods in production.	4.31%	7.08%	10.00%	18.15%	24.62%	18.31%	17.54%

* Likert scale: (1) Strongly Disagree; (2) Disagree; (3) Somewhat Disagree; (4) Neither Agree Nor Disagree; (5) Somewhat Agree; (6) Agree; (7) Strongly Agree.

**Table 7:** Knowledge of green food products—response rates (N = 650).

Statements/Scale *	1	2	3	4	5	6	7
I have knowledge about green food.	2.77%	11.54%	18.62%	46.01%	15.54%	5.23%	0.30%
I have knowledge of environmentally friendly production methods.	2.77%	6.00%	13.23%	22.31%	25.85%	17.08%	12.77%
I have knowledge about certification and labeling of green food.	11.08%	18.62%	29.69%	18.46%	10.31%	6.62%	11.08%
I have knowledge about the availability of green food.	19.08%	12.31%	8.00%	16.46%	26.31%	19.08%	12.31%

* Likert scale: (1) Strongly Disagree; (2) Disagree; (3) Somewhat Disagree; (4) Neither Agree Nor Disagree; (5) Somewhat Agree; (6) Agree; (7) Strongly Agree.

**Table 8:** Past behaviour—response rates (N = 650).

Statements/Scale *	1	2	3	4	5	6	7
I have bought green food at least once a week in the last 3 months.	18.31%	10.46%	15.85%	13.08%	19.85%	14.0%	8.46%
I have extensive experience with purchasing for green food.	1.08%	1.85%	5.23%	13.69%	20.15%	20.00%	38.00%
I am satisfied with my past purchases of green food.	17.38%	20.92%	26.92%	12.46%	5.38%	3.23%	17.38%

* Likert scale: (1) Strongly Disagree; (2) Disagree; (3) Somewhat Disagree; (4) Neither Agree Nor Disagree; (5) Somewhat Agree; (6) Agree; (7) Strongly Agree.

**Table 9:** Intentions to purchase green food products—response rates (N = 650).

Statements/Scale *	1	2	3	4	5	6	7
I plan to buy green food products in the next 3 months.	17.69%	14.77%	14.92%	14.77%	13.85%	10.46%	13.54%
I am sure that during my next purchasing I will buy an green food product. even if it has a higher price.	12.77%	13.69%	18.31%	16.00%	12.15%	14.77%	12.77%
I am willing to pay a higher price for an green product.	6.46%	10.31%	11.69%	19.85%	26.15%	12.31%	13.23%
I am willing to switch to the ecological version of the product. but if the price and quality are similar.	3.54%	4.31%	9.85%	17.08%	21.08%	40.15%	3.54%

* Likert scale: (1) Strongly Disagree; (2) Disagree; (3) Somewhat Disagree; (4) Neither Agree Nor Disagree; (5) Somewhat Agree; (6) Agree; (7) Strongly Agree.

**Table 10:** Matrix of Pearson’s correlations between independent features (TPB components and additional variables)—the extended model.

Constructs	AGFP	SN	PCB	IGFP	PB	TGFP	KGFP
AGFP	1	0.45	0.05	0.60	0.42	0.53	0.43
SN	0.45	1	0.15	0.50	0.42	0.38	0.41
PCB	0.05	0.15	1	0.21	0.27	0.19	0.34
IGFP	0.60	0.50	0.21	1	0.61	0.49	0.52
PB	0.42	0.42	0.27	0.61	1	0.39	0.49
TGFP	0.53	0.38	0.19	0.49	0.39	1	0.55
KGFP	0.43	0.41	0.34	0.52	0.49	0.55	1

**Table 11:** Regression results for “Purchase Intention” dependent variable—extended model.

Specification	b*	Std. Err. b*	b	Std. Err. b	t	*p*-Value
Intercept			3.113	0.669	4.657	0.000004 ***
PB (X1)	0.338	0.032	0.586	0.055	10.647	0.000000 ***
AGFP (X2)	0.310	0.033	0.337	0.036	9.357	0.000000 ***
SN (X3)	0.146	0.031	0.265	0.057	4.695	0.000003 ***
KGFP (X4)	0.120	0.034	0.175	0.050	3.505	0.000488 ***
TGFP (X5)	0.067	0.034	0.123	0.062	1.971	0.049157 *

* *p* < 0.05—there is a statistically significant relationship; *** *p* < 0.001—there is a very highly statistically significant relationship.

## Data Availability

Data is contained within the article.
